# The influence of the positional relationship between the pedicle and the pars interarticularis on unilateral biportal endoscopy: A retrospective cohort study

**DOI:** 10.1097/MD.0000000000047945

**Published:** 2026-03-20

**Authors:** Shaoning Shen, Tingyuan Lai, Hao Wei, Wangnan Mao, Lianguo Wu, Hanbing Zeng

**Affiliations:** a Department of Orthopaedics, The Second Affiliated Hospital of Zhejiang Chinese Medical University, Hangzhou, Zhejiang Province, China.

**Keywords:** decompression boundary, lumbar spinal stenosis, surgical efficacy, unilateral biportal endoscopy

## Abstract

This study aims to investigate the positional relationship between the pars interarticularis and the pedicle in lumbar spinal stenosis patients and clarify its guiding significance for individualized decompression strategies in unilateral biportal endoscopy (UBE). All patients received standardized UBE. DLM, laminar abduction angle [LAA], laminar width (LW), and facet joint angle (FJA) differed significantly among groups (all *P* < .001), with smaller DLM associated with narrower LW, smaller LAA, and higher FJA sagittalization. The small DLM group had lower inferior articular process reservation (24.84 ± 16.71%) and higher grade 2 to 3 destruction (75.0%, *P* < .001), accompanied by worse postoperative back visual analogue scale (VAS), Oswestry Disability Index, longer hospital stay, and greater drainage volume (all *P* < .05). No significant differences were found in dural sac area improvement or leg VAS among groups (all *P* > .05). DLM is closely associated with UBE clinical outcomes and an important anatomical reference. Smaller DLM may increase intraoperative facet joint injury risk, potentially due to compact spinal anatomy, which may worsen postoperative recovery. Preoperative DLM evaluation may help identify high-risk patients and guide individualized strategies, balancing decompression efficacy and spinal stability.

A retrospective cohort study included patients with L3-S1 lumbar spinal stenosis who underwent UBE decompression between January 2020 and December 2024. Inclusion criteria: confirmed imaging diagnosis, typical symptoms consistent with imaging, ineffective conservative treatment for ≥3 months, limited surgical segments, complete clinical/imaging data, follow-up ≥3 months. Exclusion criteria: lumbar spondylolisthesis (Meyerding grade ≥ II), prior same-segment spinal surgery, pathological stenosis, severe systemic/mental illnesses, blurred imaging data. Patients were grouped by the distance from the lateral margin of pars interarticularis to medial margin of pedicle (DLM). Evaluated indicators: preoperative imaging parameters (LAA, LW, FJA), surgical indicators, clinical outcomes (preoperative/postoperative VAS, preoperative/3-month postoperative Oswestry Disability Index, 3-month postoperative Macnab score), and postoperative imaging parameters (inferior articular process reserved amount, destruction grade, dural sac area). Statistical analyses used SPSS 26.0: ANOVA/Kruskal-Wallis H test, χ^2^ test/Fisher’s exact test, Spearman correlation, Jonckheere–Terpstra test, and ICC for consistency; *P* < .05 was significant.

## 1. Background

Lumbar spinal stenosis is a common degenerative lumbar disease in the elderly population. It is caused by hyperplasia of bony structures and hypertrophy of soft tissue structures in the spinal canal, lateral recess, or neural foramen, leading to compression of nerve roots or cauda equina. This compression causes symptoms such as low back and leg pain, intermittent claudication, and in severe cases, decreased lower extremity muscle strength and dysfunction of bowel and bladder, significantly reducing patients’ quality of life.^[1]^ For patients with ineffective conservative treatment, surgical decompression is the main method to relieve nerve compression and improve symptoms.^[2]^

With the development of minimally invasive spinal surgery techniques, unilateral biportal endoscopy (UBE) has become an important option for decompression surgery in lumbar spinal stenosis due to its advantages of clear visualization, flexible operation, and minimal trauma.^[3]^ Compared with traditional open surgery, UBE can achieve precise decompression while preserving more posterior spinal structures, showing significant advantages in reducing postoperative pain and accelerating patient recovery, and has been widely used in clinical practice.^[4]^ The core of UBE surgery for lumbar spinal stenosis lies in balancing complete decompression and structural protection,^[5]^ on the one hand, it is necessary to fully relieve the compression of nerve roots by bony and soft tissue structures in the lateral recess and neural foramen; on the other hand, excessive resection of posterior spinal stabilizing structures such as facet joints and pars interarticularis should be avoided to maintain lumbar segmental stability and reduce the risk of complications such as postoperative vertebral instability and spondylolisthesis.^[6]^ In clinical practice, the definition of decompression range often relies on anatomical landmarks. Among them, the medial margin of the pedicle, due to its constant position and easy intraoperative identification, is widely used as the lateral reference boundary for lateral recess decompression to guide surgeons in controlling the lateral extent of decompression.^[7]^

However, in clinical application of this reference boundary, it has been found that some patients experience facet joint damage after surgery, manifested as excessive resection of the inferior articular process bone, articular surface injury, and even postoperative lumbar instability. Further observation revealed that this phenomenon is closely related to the spatial relationship between the medial margin of the pedicle and the lateral margin of the pars interarticularis: as a key structure connecting the pedicle and the inferior articular process, the pars interarticularis has significant individual variations in the relative position between its lateral margin and the medial margin of the pedicle – they may completely overlap in some patients, while there are different distances between them in others.^[8]^ When the lateral margin line of the pars interarticularis overlaps with the medial margin line of the pedicle, decompression operations bounded by the medial margin of the pedicle are prone to involve the pars interarticularis and the connected inferior articular process, resulting in facet joint damage; moreover, the integrity of the pars interarticularis and facet joints is crucial for maintaining posterior spinal column stability, and their injury directly affects surgical efficacy and postoperative recovery.

Currently, existing studies mainly focus on the overall efficacy and technical optimization of UBE surgery,^[9-11]^ with few studies on the correlation between decompression reference boundaries and individual anatomical variations. Although some literatures recognize the value of the medial margin of the pedicle as a decompression reference,^[12,13]^ there is a lack of systematic analysis on decompression risks under different pedicle-pars relationships, and no personalized decompression strategy based on their spatial relationship has been established. Clinical cases of facet joint damage caused by insufficient evaluation of such anatomical variations indicate the need to further explore the impact of the relationship between the medial margin of the pedicle and the lateral margin of the pars interarticularis on decompression operations.

Therefore, this study aims to analyze the spatial relationship between the lateral margin line of the pars interarticularis and the medial margin line of the pedicle in preoperative imaging of patients with lumbar spinal stenosis, combined with intraoperative facet joint damage and postoperative efficacy, to clarify the guiding significance of this relationship for UBE surgical decompression strategies, and to provide clinical evidence for optimizing surgical procedures and reducing postoperative complications.

## 2. Materials and methods

### 2.1. Study design and ethical approval

This is a retrospective cohort study, which included patients diagnosed with lower lumbar (L3-S1) spinal stenosis who underwent UBE surgery in the Department of Orthopedics, the Second Affiliated Hospital of Zhejiang Chinese Medical University from January 2020 to December 2024. This study was approved by the Ethics Review Committee of the second Affiliated Hospital of Zhejiang Chinese Medicine University and obtained the unique identification number of research registration (Ethics Approval No.: 2025-189-01). Each patient signed a written informed consent form. In this study, all methods were performed in accordance with the Declaration of Helsinki relevant guidelines and regulations.

### 2.2. Study subjects

#### 2.2.1. Inclusion criteria

All imaging parameters were measured based on preoperative lumbar CT and MRI images. Imaging (CT/MRI) confirmed the presence of spinal stenosis at the L3-S1 segments; typical clinical manifestations of low back and leg pain, intermittent claudication, with symptoms consistent with the stenotic segments shown by imaging; ineffective conservative treatment (including medication, physical therapy, etc) for ≥3 months; underwent UBE surgery with the surgical segments limited to L3-S1;and complete clinical and imaging data, with a postoperative follow-up duration of ≥3 months.

#### 2.2.2. Exclusion criteria

Lumbar spondylolisthesis (Meyerding grade ≥ Ⅱ), spondylolysis, or spinal instability; a history of spinal surgery at the same segments; pathological spinal stenosis caused by tumors, infections, fractures, etc; complicated with severe systemic diseases (such as heart, liver, or kidney diseases) or mental illnesses, unable to cooperate with follow-up;and blurred imaging data that prevented measurement of relevant imaging parameters.

### 2.3. Grouping method and criteria

All patients underwent preoperative lumbar anteroposterior and lateral X-rays, lumbar CT, and MRI. Two senior orthopedic surgeons (with working experience ≥5 years) independently measured the relative relationship between the lateral margin line of the pars interarticularis and the medial margin line of the pedicle using a blind method (without being informed of patients’ clinical information). If there were discrepancies between the 2 measurement results, a third chief physician would review and confirm the final result. Group 1: the lateral margin line of the pars interarticularis is located medial to or overlaps with the medial margin line of the pedicle. (distance ≤ 0 mm); Group 2: the lateral margin line of the pars interarticularis is located between the medial margin line of the pedicle and the midpoint of the pedicle (0 mm < distance ≤ 1/2 of the pedicle width); Group 3: the lateral margin line of the pars interarticularis is located beyond the midpoint of the pedicle (distance > 1/2 of the pedicle width).

### 2.4. Observation indicators

#### 2.4.1. Baseline data

The following data were collected: gender, age, height, weight, body mass index (BMI = weight/height^2^, kg/m^2^), follow-up duration, surgical segments (L3/4, L4/5, L5/S1), and surgical side (left/right).

#### 2.4.2. Imaging parameters

**Figure 1.**
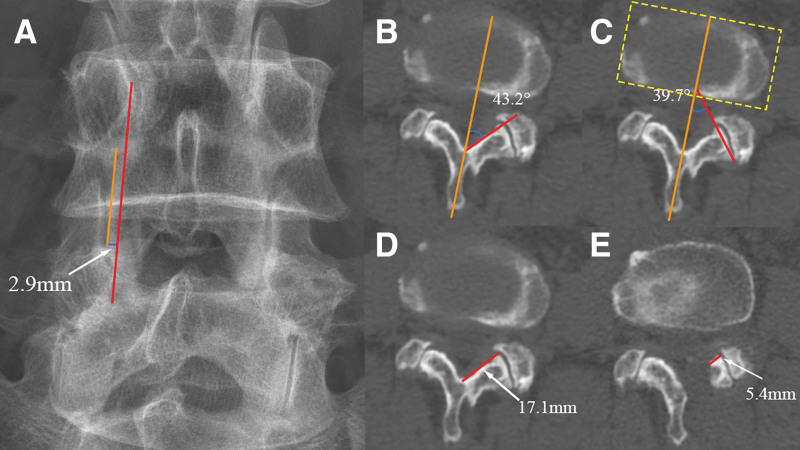
Examples of imaging measurements. (A) The red line represents the medial connecting line of the pedicle. The orange line represents the lateral margin line of the pars interarticularis, and the blue line represents the vertical distance between them, which is DLM. (B) The orange line represents the central axis of the spinous process, and the red line represents the medial margin line of the inferior articular process; the angle formed between them is LAA. (C) The orange line represents the midline of the intervertebral disc, and the red line represents the connecting line between the medial and lateral margins of the facet joint surface; the angle formed between them is FJA. (D) The shortest distance from the base of the spinous process to the lateral margin of the ipsilateral lamina is measured as the preoperative laminar width. (E) The remaining width of the inferior articular process is measured postoperatively. The ratio of this remaining width to the preoperative laminar width is the reserved amount of the inferior articular process. DLM = distance from the lateral margin line of the pars interarticularis to the medial margin line of the pedicle, LAA = laminar abduction angle, FJA = facet joint angle.

**Figure 2.**
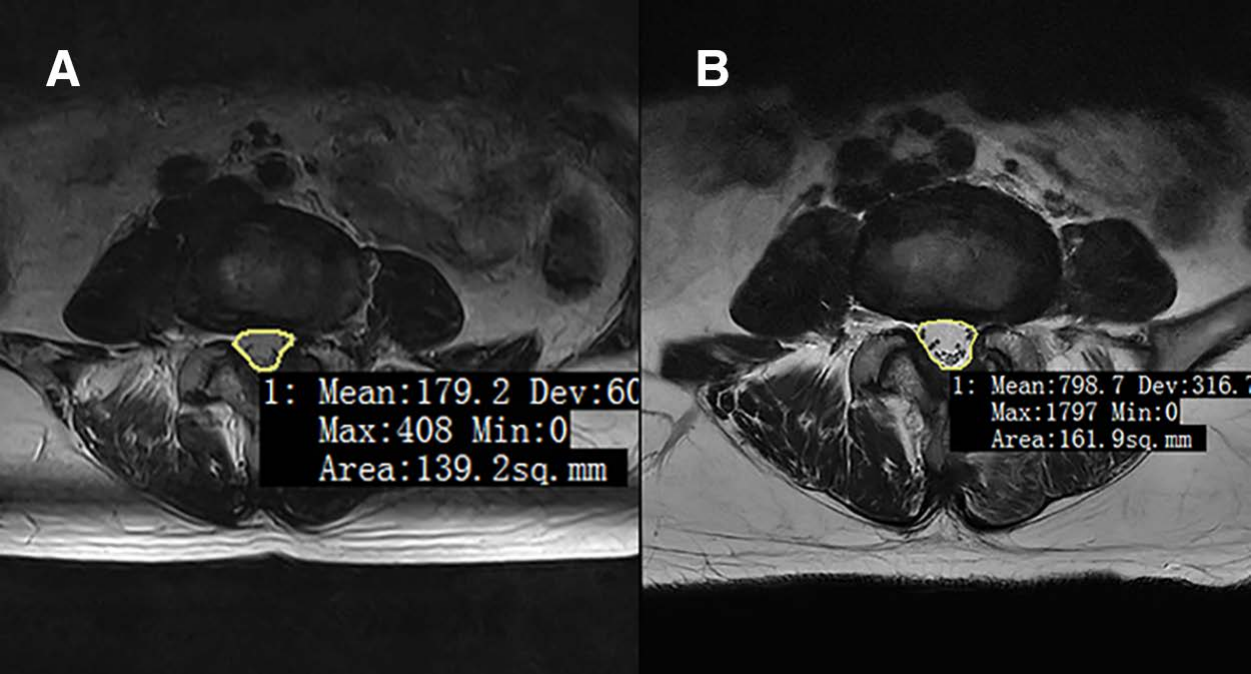
Measurement of dural sac area. (A) Preoperative and (B) postoperative.

The distance from the lateral margin line of the pars interarticularis to the medial margin line of the pedicle (DLM): the direct measurement value for the above grouping (mm), with negative values indicating the position within the medial margin line of the pedicle and positive values indicating the position outside; Laminar abduction angle (LAA): on the cross-section passing through the base of the spinous process and parallel to the lower endplate of the upper vertebra, with the central axis of the spinous process as the reference line, the angle between the “line from the base of the spinous process to the medial margin of the ipsilateral inferior articular process” and the central axis of the spinous process was measured; Laminar width (LW): on the same plane as the measurement of LAA, the shortest distance from the base of the spinous process to the lateral margin of the ipsilateral lamina was measured; Facet joint angle (FJA): with the midline of the intervertebral disc as the sagittal reference line, the angle between the line connecting the medial and lateral margins of the unilateral facet joint surface and the sagittal plane was measured^[14]^; Reserved amount of the inferior articular process: the ratio (%) of the remaining width of the inferior articular process measured on postoperative coronal CT to the preoperative LW; Grade of inferior articular process destruction^[15]^: classified into 4 grades according to the degree of destruction – Grade 0: minimal destruction (destruction range < 2 mm); Grade 1: mild damage to the inferior articular process (destruction range < 1/2); Grade 2: moderate destruction (destruction range ≥ 1/2 but not complete); Grade 3: severe destruction (complete destruction); Dural sac area: the maximum cross-sectional area of the dural sac on MRI T2-weighted images at the surgical segment before surgery and 3 months after surgery (mm^2^). Examples of measurements are shown in Figures 1 and 2.

#### 2.4.3. Surgical efficacy indicators

Surgery-related indicators: operation duration (min), drainage volume through the drainage tube within 24 hours after surgery (mL), length of hospital stay (day); The visual analogue scale (VAS) was used to evaluate the degree of low back and leg pain before surgery, 1 day after surgery, and 3 days after surgery. The Oswestry Disability Index (ODI) was used to assess lumbar function before surgery and 3 months after surgery; the Macnab score was used to evaluate patient satisfaction 3 months after surgery.

### 2.5. Surgical technique

**Figure 3.**
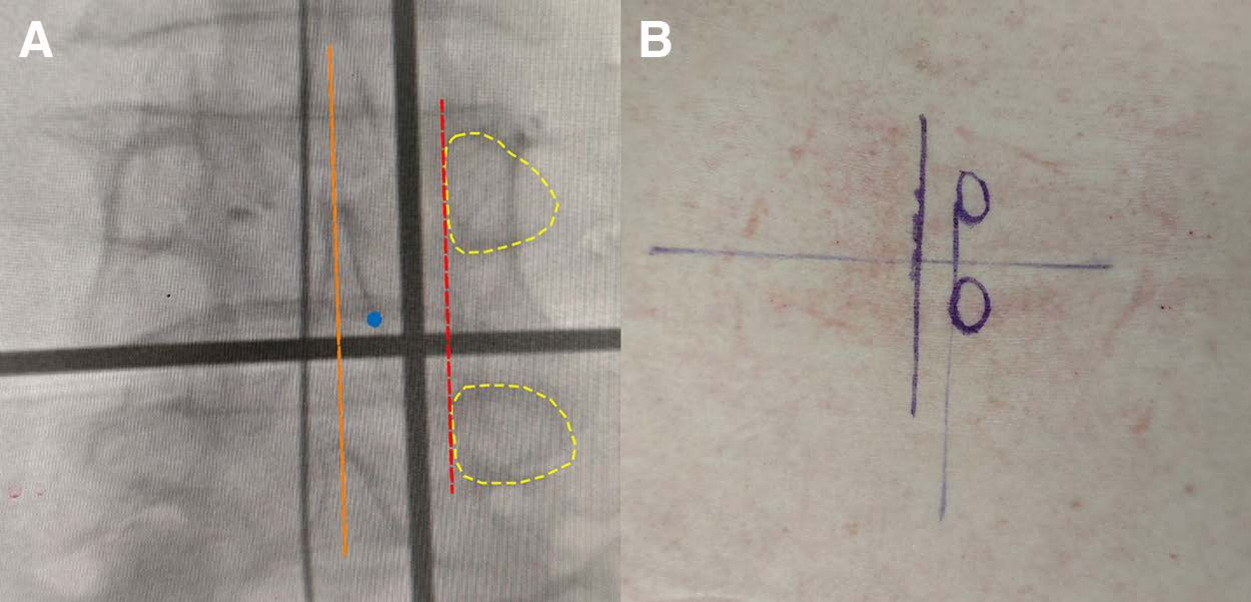
Preoperative X-ray localization and body surface marking. (A) Anteroposterior-X-ray fluoroscopy image of the surgical lumbar segment before operation, with Kirschner wires placed on the body surface for marking. The thin longitudinal Kirschner wire (orange line) indicates the spinous process line; the thick Kirschner wire (red line) indicates the line of the medial edge of the pedicle (all Kirschner wires are placed slightly to the left to avoid obscuring anatomical structures). The transverse Kirschner wire indicates the intervertebral space level; the blue dot indicates the initial point; the yellow line represents the pedicle contour. (B) Corresponding markings made on the body surface based on the preoperative fluoroscopy images.

All surgeries were performed by the same team of experienced physicians (with ≥200 UBE surgery cases) using standard UBE techniques: After general anesthesia, the patient was placed in the prone position. The responsible intervertebral space was confirmed via C-arm fluoroscopy, and the spinous process-lamina junction on the anteroposterior view was marked as the initial operation point. The medial margin line of the pedicle (the connecting line of the medial margins of bilateral pedicles) was marked as the reference, and 2 parallel incisions were made along this line. The cephalad incision was a 5 to 6 mm diameter observation channel incision, located above the initial point; the caudal incision was an 8 to 10 mm diameter working channel incision, 2.5 to 3 cm away from the observation channel to ensure no conflict in instrument operation. Figure 3 shows an example of intraoperative fluoroscopic localization. A 4 mm 30° endoscope was inserted through the observation channel, and a dilator was inserted through the working channel. The multifidus muscle was bluntly dissected to the lamina surface to avoid excessive muscle traction. Continuous saline irrigation was connected (pressure 30–40 mm Hg) to remove soft tissues through water flow, clearly exposing the spinous process-lamina junction and the inferior edge of the lamina. Starting from the spinous process-lamina junction, a 3 to 4 mm high-speed drill was used to remove part of the bone at the inferior edge of the lamina, exposing the attachment site of the ligamentum flavum. The superficial ligamentum flavum was first resected to obtain a clear visual field, while the deep ligamentum flavum was retained as a barrier to protect the dural sac and nerve roots, preventing damage caused by direct contact of the drill or instruments with neural tissues. Focus was placed on the junction of the medial pedicle and the superior articular process (corner area), where the drill was used to gradually remove the hyperplastic bone until the medial margin of the pedicle was clearly exposed as the lateral boundary of decompression. Along the exposed medial margin of the pedicle, the medial bone of the superior articular process was further drilled upward, and osteophytes on the medial margin of the pedicle were removed. After confirming the range of bony decompression, lamina rongeurs were used to gradually separate and resect the deep ligamentum flavum from the edge to the center, completely relieving the compression on the nerve roots. Small vessel bleeding was coagulated with radiofrequency, and bone surface oozing was sealed with bone wax. A drainage tube was placed, the channels were removed, and the incisions were sutured; the drainage tube was usually removed after 24 hours.

### 2.6. Statistical analysis

SPSS 26.0 software (Chicago) was used for data analysis. Measurement data conforming to a normal distribution were expressed as mean ± standard deviation. Comparisons among multiple groups were performed using 1-way analysis of variance (ANOVA), with pairwise comparisons between groups using the Bonferroni correction test. Measurement data not conforming to a normal distribution were expressed as median (interquartile range), and comparisons among groups were performed using the Kruskal–Wallis H test, with pairwise comparisons using the Bonferroni-corrected rank sum test. Count data were expressed as frequencies, and comparisons between groups were performed using the χ^2^ test or Fisher’s exact test. Spearman rank correlation analysis was used to evaluate the correlation between variables, and the Jonckheere–Terpstra test was used to analyze the trend of ordered grouped data. The ICC was used to assess the consistency of measurement results between the 2 physicians, with an ICC > 0.75 indicating good consistency. *P* value < .05 was considered statistically significant.

## 3. Results

**Figure 4.**
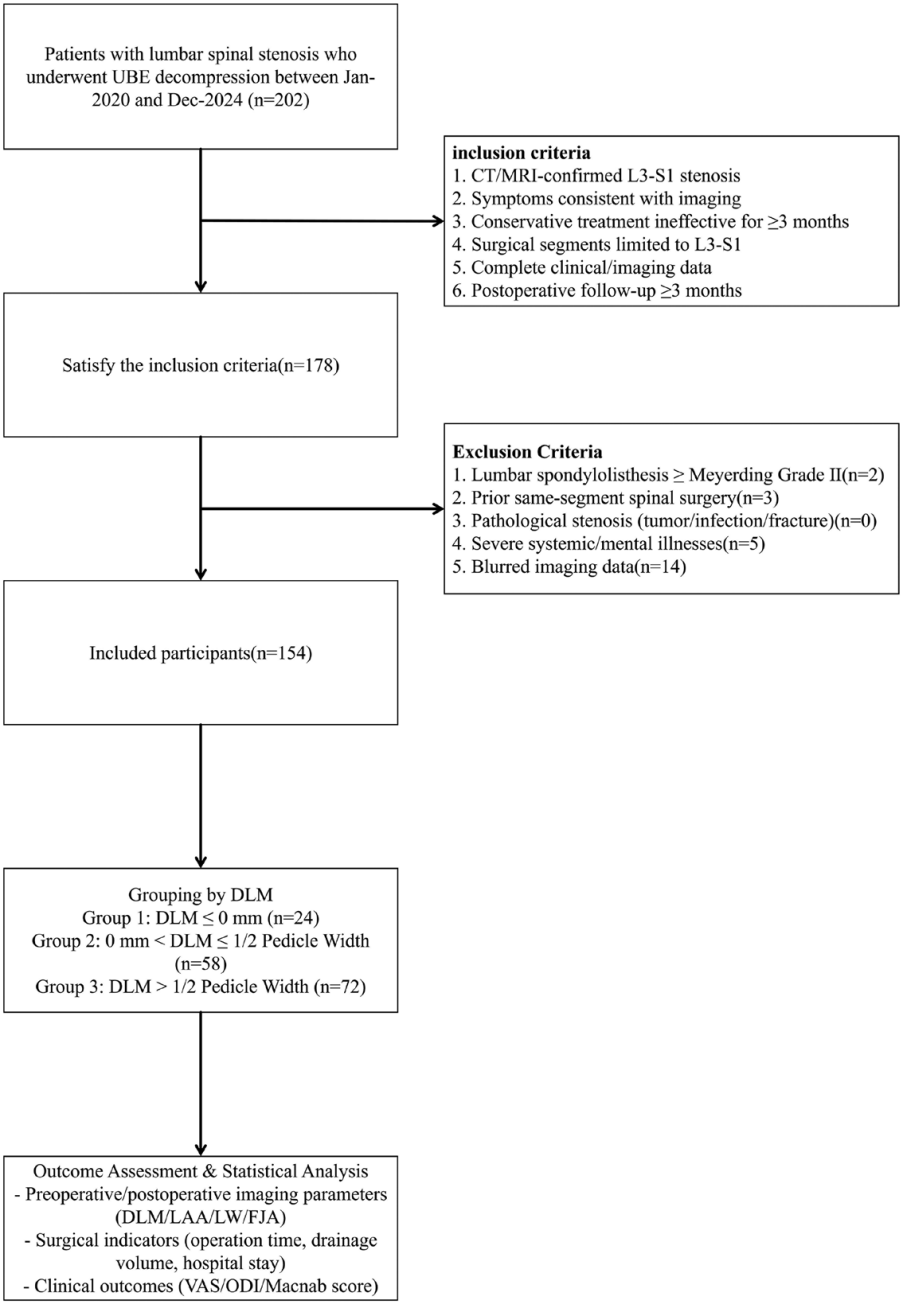
Flowchart of the enrolled cases. CT = computed tomography, DLM = distance from the lateral margin line of the pars interarticularis to the medial margin line of the pedicle, MRI = magnetic resonance imaging, UBE = unilateral biportal endoscopy.

From January 2020 to December 2024, a total of 202 patients with lumbar spinal stenosis underwent UBE decompression at the Second Affiliated Hospital of Zhejiang Chinese Medical University. Among them, 178 patients met the inclusion criteria. After screening against the exclusion criteria, 154 patients were finally enrolled. These enrolled patients were divided into 3 groups based on the DLM: group with DLM ≤ 0 mm (24 cases), group with 0 mm < DLM ≤ 1/2 pedicle width (58 cases), and group with DLM > 1/2 pedicle width (72 cases). Details are shown in Figure 4.

There were no statistically significant differences in gender, age, body mass index, surgical side, or follow-up duration among the 3 groups of patients included in this study (all *P* > .05), indicating they were comparable. However, there was a statistically significant difference in the distribution of surgical segments among the 3 groups (*P* < .001), and pairwise comparisons between groups also showed statistical differences. Specifically, group 1 had a high proportion of L3/4 segment surgeries (66.7%, 16/24) with no L5/S1 segment cases, while group 2 was dominated by L4/5 segment surgeries (63.8%, 37/58) and only a small proportion of L5/S1 cases (12.1%, 7/58). Group 3 had the highest proportion of L5/S1 segment surgeries (41.7%, 30/72), with L4/5 segment surgeries accounting for 55.6% (40/72) and L3/4 segment surgeries only 2.8% (2/72). Details are shown in Table 1.

**Table 1:** Baseline data of patients in the 3 groups.

Variable	Group 1	Group 2	Group 3	*F*/χ^2^	*P* value
Gender (male, female)	10 (41.7%), 14 (58.3%)^*^	32 (55.2%), 26 (44.8%)^*^	42 (58.3%), 30 (41.7%)^*^	2.031	.362
Age (yr)	54.25 ± 8.76^*^	57.84 ± 11.54^*^	56.79 ± 11.58^*^	0.877	.418
BMI	21.80 ± 0.74^*^	21.94 ± 1.08^*^	22.02 ± 1.38^*^	0.337	.715
Surgical segment (L3/4, L4/5, L5/S1)	16 (66.7%), 8 (33.3%), 0 (0.0%)^*^	14 (24.1%), 37 (63.8%), 7 (12.1%)^†^	2 (2.8%), 40 (55.6%), 30 (41.7%)^‡^	57.263	<.001
Operative side (left, right)	17 (70.8%), 7 (29.2%)^*^	30 (51.7%), 28 (48.3%)^*^	37 (51.4%), 35 (48.6%)^*^	3.043	.218
Follow-up duration (mo)	8.88 ± 1.85^*^	8.66 ± 1.9^*^	9.11 ± 2.07^*^	0.853	.428

* /†/‡: Superscripts with different symbols indicate that there are statistically significant differences in post hoc comparisons, while the same symbols indicate no statistically significant differences.

BMI = body mass index.

In terms of anatomical parameters, there were statistically significant differences in DLM, LAA, LW, and FJA among the 3 groups of patients (all *P* < .001). Specifically, DLM was 1.38 ± 1.13 mm in group 1, 5.58 ± 0.82 mm in group 2, and 7.55 ± 1.76 mm in group 3; LAA was 39.71 ± 2.00° in group 1, 44.35 ± 3.21° in group 2, and 53.81 ± 5.43° in group 3; LW was 12.65 ± 1.08 mm in group 1, 14.30 ± 2.52 mm in group 2, and 16.68 ± 2.77 mm in group 3; FJA was 40.43 ± 3.86° in group 1, 46.17 ± 5.52° in group 2, and 55.95 ± 9.58° in group 3. Correlation analysis and trend test showed that the group grading was significantly correlated with these 4 parameters, presenting a gradual increasing trend from group 1 to group 3. Details are shown in Tables 2 and 3.

**Table 2:** Spinal imaging parameters of patients in the 3 groups.

Variable	Group 1	Group 2	Group 3	*F*	*P* value
DLM (mm)	1.38 ± 1.13^*^	5.58 ± 0.82^†^	7.55 ± 1.76^‡^	181.682	<.001
LAA (°)	39.71 ± 2.00^*^	44.35 ± 3.21^†^	53.81 ± 5.43^^‡^^	132.006	<.001
LW (mm)	12.65 ± 1.08^*^	14.30 ± 2.52^†^	16.68 ± 2.77^‡^	29.068	<.001
FJA (°)	40.43 ± 3.86^*^	46.17 ± 5.52^†^	55.95 ± 9.58^‡^	49.109	<.001

* /†/‡: Superscripts with different symbols indicate that there are statistically significant differences in post hoc comparisons, while the same symbols indicate no statistically significant differences.

DLM = distance from the lateral margin line of the pars interarticularis to the medial margin line of the pedicle, FJA = facet joint angle, LAA = laminar abduction angle, LW = laminar width.

**Table 3:** Correlation and trend test of DLM grouping grades among the 3 groups of patients.

Variable	Spearman’s *P*	*P* value	J-T’ Z value	*P* value
DLM (mm)	.756	<.001	10.086	<.001
LAA (°)	.833	<.001	11.015	<.001
LW (mm)	.533	<.001	6.639	<.001
FJA (°)	.632	<.001	8.153	<.001

DLM = distance from the lateral margin line of the pars interarticularis to the medial margin line of the pedicle, FJA = facet joint angle, LAA = laminar abduction angle, LW = laminar width.

Results regarding the dural sac area and articular process preservation showed that there were no statistically significant differences in the preoperative dural sac area, postoperative dural sac area, or the difference between the 2 among the 3 groups (all *P* > .05). There was a significant difference in the reserved amount of the inferior articular process among the 3 groups (*P* < .001), showing a gradual increasing trend from group 1 to group 3: the mean reserved rate was 24.84 ± 16.71% in group 1, 73.58 ± 14.56% in group 2, and 88.79 ± 8.22% in group 3. Additionally, there was a significant difference in the grade of inferior articular process destruction among the 3 groups (*P* < .001), with statistically significant differences in pairwise comparisons between groups. Specifically, group 1 had the highest proportion of severe destruction (Grade 2–3: 70.8% [17/24] and 29.2% [7/24], respectively) and no cases of minimal or mild destruction (Grade 0–1). Group 2 was dominated by mild destruction (Grade 1: 86.2% [50/58]), with only 13.8% (8/58) of cases showing moderate destruction (Grade 2) and no severe destruction. Group 3 mostly had minimal destruction (Grade 0: 70.8% [51/72]) or mild destruction (Grade 1: 27.8% [20/72]), with only 1.4% (1/72) of cases having moderate destruction and no severe destruction. Different groups presented distinct distribution characteristics of destruction degrees. Details are shown in Table 4.

**Table 4:** Postoperative imaging evaluation of patients in the 3 groups.

Variable	Group 1	Group 2	Group 3	*F*	*P* value
Pre-DSA (mm^2^)	127.47 ± 16.95^*^	123.55 ± 14.46^*^	129.63 ± 14.64^*^	2.677	.072
Po-DSA (mm^2^)	173.83 ± 26.34^*^	168.09 ± 22.56^*^	175.13 ± 22.09^*^	1.573	.211
DSA difference (mm^2^)	46.36 ± 14.29^*^	44.54 ± 11.33^*^	45.49 ± 11.77^*^	0.216	.806
Facet joint preservation distance (%)	24.84 ± 16.71^*^	73.58 ± 14.56^†^	88.79 ± 8.22^‡^	238.449	<.001
Facet joint preservation grade (0, 1, 2, 3)	0 (0.0%), 0 (0.0%), 17 (70.8%), 7 (29.2%)^*^	0 (0.0%), 50 (86.2%), 8 (13.8%), 0 (0.0%)^†^	51 (70.8%), 20 (27.8%), 1 (1.4%), 0 (0.0%)^‡^	184.991	<.001

* /†/‡: Superscripts with different symbols indicate that there are statistically significant differences in post hoc comparisons, while the same symbols indicate no statistically significant differences.

DSA = dural sac area.

In terms of clinical function and surgical indicators, there were no statistically significant differences in preoperative ODI and preoperative back VAS among the 3 groups (*P* > .05). There were statistically significant differences in postoperative ODI, back VAS on the first day after surgery, and back VAS at 3 months after surgery among the 3 groups (*P* < .001), with group 1 being significantly higher than group 2 and group 3 (no difference between the latter 2 groups). Specifically, the mean postoperative ODI was 23.04 ± 5.03 in group 1, 18.50 ± 4.58 in group 2, and 18.78 ± 4.75 in group 3; the mean back VAS on the first day after surgery was 2.92 ± 0.65 in group 1, 2.07 ± 0.92 in group 2, and 2.01 ± 0.96 in group 3; the mean back VAS at 3 months after surgery was 2.63 ± 0.92 in group 1, 1.53 ± 1.19 in group 2, and 1.46 ± 1.33 in group 3.

There were no statistically significant differences in leg VAS before surgery, on the first day after surgery, or at 3 months after surgery among the 3 groups (*P* > .05). There were statistically significant differences in hospital stay and postoperative drainage volume among the 3 groups (*P* < .001), with group 1 being significantly higher than group 2 and group 3: the mean length of hospital stay was 128.13 ± 37.06 hours in group 1, 110.45 ± 21.50 hours in group 2, and 109.96 ± 21.79 hours in group 3; the mean postoperative drainage volume was 42.17 ± 8.95 mL in group 1, 29.21 ± 11.52 mL in group 2, and 25.89 ± 11.08 mL in group 3.

There was no statistically significant difference in operation time among the 3 groups (*P* = .340), and there was no statistically significant difference in Macnab scores among the 3 groups (*P* = .558) – group 1 had 16 cases (66.7%) with good outcomes and 8 cases (33.3%) with excellent outcomes; group 2 had 39 cases (67.2%) with good outcomes and 19 cases (32.8%) with excellent outcomes; group 3 had 54 cases (75.0%) with good outcomes and 18 cases (25.0%) with excellent outcomes, showing consistent favorable outcome distributions across groups. Details are shown in Table 5.

**Table 5:** Clinical efficacy evaluation of the 3 groups of patients.

Variable	Group 1	Group 2	Group 3	*F*/χ^2^	*P* value
pre-ODI	49.50 ± 5.99^*^	49.74 ± 6.19^*^	50.17 ± 5.70^*^	0.148	.863
po-ODI	23.04 ± 5.03^*^	18.50 ± 4.58^†^	18.78 ± 4.75^†^	8.784	<.001
Pre- back vas	5.83 ± 1.58^*^	5.91 ± 1.41^*^	6.17 ± 1.29^*^	1.602	.449
Po-1day back vas	2.92 ± 0.65^*^	2.07 ± 0.92^†^	2.01 ± 0.96^†^	17.158	<.001
Po-3months back vas	2.63 ± 0.92^*^	1.53 ± 1.19^†^	1.46 ± 1.33^†^	18.969	<.001
Pre-leg vas	6.88 ± 1.54^*^	6.98 ± 1.35*	7.08 ± 1.24^*^	0.243	.785
Po-1day leg vas	1.29 ± 0.99^*^	1.50 ± 1.59^*^	1.32 ± 1.06^*^	0.393	.676
Po-3months leg vas	0.96 ± 0.69^*^	0.83 ± 0.91^*^	0.99 ± 0.87^*^	0.665	.516
Hospitalization length (h)	128.13 ± 37.06^*^	110.45 ± 21.50^†^	109.96 ± 21.79^†^	5.383	.006
Operative time (min)	111.96 ± 23.58^*^	106.73 ± 19.69^*^	104.73 ± 20.71^*^	1.087	.340
Drainage volume (mL)	42.17 ± 8.95^*^	29.21 ± 11.52^†^	25.89 ± 11.08^†^	19.96	<.001
Macnab (poor, fair, good, excellent)	0 (0.0%), 0 (0.0%), 16 (66.7%), 8 (33.3%)^*^	0 (0.0%), 0 (0.0%), 39 (67.2%), 19 (32.8%)^*^	0 (0.0%), 0 (0.0%), 54 (75.0%), 18 (25.0%)^*^	1.167	.558

* /†: Superscripts with different symbols indicate that there are statistically significant differences in post hoc comparisons, while the samesymbols indicate no statistically significant differences.

ODI = Oswestry Disability Index.

## 4. Discussion

UBE technology has become an important treatment option for lumbar spinal stenosis due to its advantages of clear visualization, flexible operation, and sufficient decompression. Its learning curve is relatively gentle, and an increasing number of spinal surgeons, especially young physicians, have begun to adopt this technique.^[16]^ However, the definition of decompression range in UBE surgery relies on anatomical landmarks such as the medial edge of the pedicle. If novice surgeons blindly follow a unified lateral boundary for decompression and ignore individual anatomical variations, they may easily cause excessive damage to the facet joints.

In this study, by analyzing the spatial distance (DLM) between the lateral margin line of the pars interarticularis and the medial margin line of the pedicle in preoperative imaging of patients with lumbar spinal stenosis, we found significant differences among the 3 groups in anatomical parameters, facet joint preservation and destruction, and clinical efficacy indicators. Anatomically, DLM, LAA, LW, and FJA showed a gradual increasing trend across the 3 groups. Specifically, the small DLM group (group 1) had smaller values of LAA, LW, and FJA, indicating that the positions of the pars interarticularis, facet joints, and pedicles are highly concentrated; in contrast, the large DLM group (group 3) showed more dispersed anatomical structures.

Regarding the preservation and destruction of facet joints, group 1 had the lowest reserved amount of inferior facet joints, with 100% of cases showing grade 2 or higher destruction; group 3 had the highest reserved amount, with only 1 patient experiencing grade 2 destruction of the inferior facet joint. In terms of clinical efficacy, there were no significant differences in preoperative VAS and ODI among the 3 groups, but postoperative ODI, back VAS score on the first day after surgery, and back VAS score at 3 months after surgery in group 1 were significantly higher than those in group 2 and group 3; the length of hospital stay and postoperative drainage volume also increased significantly. Notably, there were no statistically significant differences in preoperative and postoperative dural sac area or their differences among the 3 groups, and there was no significant difference in postoperative leg VAS scores among the 3 groups (*P* > .05). This indicates that UBE surgery can achieve effective decompression regardless of DLM size,^[17]^ but the small DLM group showed poorer postoperative pain relief and functional recovery due to more severe facet joint damage.

Mechanistically, the differences in efficacy in the small DLM group are closely related to the characteristics of anatomical structures. Spatial overlap between the pars interarticularis and the pedicle increases the risk of surgical trauma.^[12]^ Group 1 had a small LAA and narrow LW, resulting in limited operating space. When using the medial edge of the pedicle as the decompression boundary, it is easy to damage the pars interarticularis and facet joints. A small FJA indicates a high degree of sagittalization of the facet joints, whose stability depends on bony structures^[14,18]^; excessive damage can exacerbate postoperative pain and dysfunction. In addition, the longer hospital stay and increased drainage volume in group 1 may be related to increased local oozing after facet joint injury, confirming the clinical rule that the more severe the structural damage, the slower the postoperative recovery. In contrast, group 3, with sufficient anatomical space, achieved adequate decompression while preserving the facet joints during decompression, resulting in milder postoperative pain, faster recovery, and no difference in dural sac area compared with other groups. This indicates that group 3 achieved a better balance between complete decompression and structural protection.

Biomechanical tests have shown that more than 50% of facet joint destruction can lead to segmental instability.^[15]^ Therefore, based on the differences in efficacy among DLM-based groups, targeted surgical strategies need to be formulated. For group 1 (small DLM, such as L3/4), since ipsilateral approach will inevitably cause damage to the facet joints and lead to postoperative instability, while contralateral approach can maintain spinal biomechanical stability by preserving more facet joints, the contralateral approach is recommended.^[19]^ During the operation, grinding 1 to 2 mm of bone at the base of the spinous process to expand the channel and using a 30° endoscope to reduce blind areas in the field of view are helpful for precise decompression without additional damage to surrounding structures, so as to improve postoperative pain and shorten hospital stay. However, it should be noted that when using a drill to enter the contralateral side, an angle larger than LAA may cause lamina penetration, and in severe cases, even transverse damage to the spinous process. Conversely, an angle smaller than LAA may increase the risk of dural sac and nerve injury.^[20]^ For group 2 (moderate DLM), preoperative precise measurement of DLM, LW and FJA by CT is required, and the ipsilateral approach can be selected. The incision is appropriately deviated to the midline to increase the operating space. During decompression, a 3 mm thin drill is used for limited resection.^[20]^ For the corner area, the medial edge of the pedicle can be used as a reference for the lateral boundary of decompression, and the upward decompression range should not exceed the tip of the superior articular process. This can balance decompression efficiency and facet joint protection. For group 3 (large DLM, such as L5/S1), the standard unilateral approach with bilateral decompression technique is adopted.^[7]^ Angled lamina rongeurs are used to deal with the lateral recess with reference to the medial edge of the pedicle. The lowest postoperative VAS, ODI and the least drainage volume in this group indicate that this strategy is safe and efficient.

In addition, attention should be paid to the impact of synergistic factors on efficacy. The superposition of small FJA and small DLM in group 1 may increase the risk of facet joint degeneration. Postoperatively, dynamic X-ray should be used to monitor spinal stability to avoid aggravated spondylolisthesis and pain during long-term follow-up. Although this study did not include patients with lumbar disc herniation, for patients with lumbar disc herniation as the main symptom, if combined with small DLM, the decompression range can be reduced to protect the facet joints,^[21]^ so as to reduce drainage volume and hospital stay.

This study excluded patients with L1/L2 and L2/3 segments because previous observations found that the lateral margin line of the pars interarticularis in these segments is mostly located within the medial margin line of the pedicle, and the probability of facet joint injury during ipsilateral approach is extremely high. From an anatomical perspective, the upper lumbar vertebrae (L1/L2, L2/3) have smaller LAA and narrower LW, resulting in limited operating space.^[22,23]^ Even with precise control of instrument angles in ipsilateral approach, excessive resection of facet joints is still difficult to avoid. Therefore, for patients with spinal stenosis in L1/L2 and L2/3 segments, it is recommended to directly adopt contralateral approach or far lateral transforaminal approach.^[24,25]^

This study has several limitations. First, the retrospective design may introduce selection bias and residual confounding, as unmeasured factors (e.g., preoperative comorbidities, intraoperative surgical technique details beyond the standardized protocol) could potentially influence the outcomes. Second, the follow-up time is relatively short (average 8–9 months), and long-term spinal stability (e.g., development of spondylolisthesis or recurrent stenosis) has not been evaluated. Third, DLM measurement relies on 2-dimensional images, which may ignore 3-dimensional anatomical variations that could affect surgical decision-making. Fourth, this study did not perform multivariable analysis to adjust for potential confounding factors (e.g., age, surgical segment distribution, which showed significant differences among groups), which limits the ability to isolate the independent effect of DLM on surgical outcomes. Fifth, the study excluded patients with L1/L2 and L2/3 segments, which may limit the generalizability of the findings to the entire lumbar spine.

## 5. Conclusion

DLM is closely associated with the clinical outcomes of UBE surgery and serves as an important anatomical reference indicator worthy of attention. A smaller DLM value indicates a closer positional relationship between the pars interarticularis and the pedicle, leading to a higher risk of intraoperative facet joint injury in patients. The potential reason for this phenomenon may be that patients with smaller DLM exhibit the characteristic of compact anatomical arrangement of spinal structures, specifically manifested as narrower LW, smaller LAA, and higher sagittalization of FJA; this anatomical feature makes it more difficult to avoid facet joint injury during UBE surgical procedures, thereby increasing the risk of injury. Moreover, secondary facet joint injury during surgery may be a potential contributor to more significant postoperative low back pain, higher degrees of functional impairment, and prolonged recovery periods in patients. Preoperative evaluation of DLM and its related anatomical parameters helps identify high-risk populations for facet joint injury and provides a reference for formulating individualized decompression strategies – such as adjusting the decompression boundary or surgical approach for high-risk patients. This further achieves a balance between ensuring the effectiveness of neural decompression and maintaining spinal segmental stability, ultimately optimizing the quality of patients’ postoperative rehabilitation.

## Author contributions

**Conceptualization:** Lianguo Wu, Hanbing Zeng.

**Data curation:** Shaoning Shen.

**Formal analysis:** Wangnan Mao, Lianguo Wu.

**Investigation:** Shaoning Shen, Wangnan Mao.

**Methodology:** Hanbing Zeng.

**Project administration:** Hanbing Zeng.

**Resources:** Tingyuan Lai.

**Software:** Tingyuan Lai, Hao Wei.

**Supervision:** Hao Wei.

## References

[1] Katz JN Zimmerman ZE Mass H Makhni MC. Diagnosis and management of lumbar spinal stenosis: a review. JAMA. 2022;327:1688–99. 35503342 10.1001/jama.2022.5921

[2] Chen L Guan B Anderson DB. Surgical interventions for degenerative lumbar spinal stenosis: a systematic review with network meta-analysis. BMC Med. 2024;22:430. 39379938 10.1186/s12916-024-03653-z PMC11463109

[3] Kim JE Choi DJ. Unilateral biportal endoscopic decompression by 30° endoscopy in lumbar spinal stenosis: technical note and preliminary report. J Orthop. 2018;15:366–71. 29881155 10.1016/j.jor.2018.01.039 PMC5990374

[4] Lee DH Lee DG Park CK. Saving stabilizing structure treatment with bilateral-contralateral decompression for spinal stenosis in degenerative spondylolisthesis using unilateral biportal endoscopy. Neurospine. 2023;20:931–9. 37798987 10.14245/ns.2346504.252 PMC10562235

[5] Pao JL Lin SM Chen WC Chang CH. Unilateral biportal endoscopic decompression for degenerative lumbar canal stenosis. J Spine Surg (Hong Kong). 2020;6:438–46. 10.21037/jss.2020.03.08 PMC7340817 32656381

[6] Pao J-L. A Review of unilateral biportal endoscopic decompression for degenerative lumbar canal stenosis. Int J Spine Surg. 2021;15(suppl 3):S65–71. 10.14444/8165 PMC9421212 35027470

[7] Hu Y Fu H Yang D Xu W. Clinical efficacy and imaging outcomes of unilateral biportal endoscopy with unilateral laminotomy for bilateral decompression in the treatment of severe lumbar spinal stenosis. Front Surg. 2023;9:1061566. 10.3389/fsurg.2022.1061566 PMC9852342 36684266

[8] Rexiti P Abulizi Y Muheremu A. Anatomical and radiologic characteristics of isthmus parameters in guiding pedicle screw placement. J Int Med Res. 2018;46:2386–97. 29619849 10.1177/0300060518762986 PMC6023064

[9] Yang L Yu T Jiao J. Comprehensive analysis of UBE-related complications: prevention and management strategies from 4685 patients. Med Sci Monitor. 2024;30:e944018. 10.12659/MSM.944018 PMC11476038 39385451

[10] Deng C Li X Wu C Xie W Chen M. One-hole split endoscopy versus unilateral biportal endoscopy for lumbar degenerative disease: a systematic review and meta-analysis of clinical outcomes and complications. J Orthop Surg Res. 2025;20:187. 39985036 10.1186/s13018-025-05591-9 PMC11844110

[11] Chu PL Wang T Zheng JL. Global and current research trends of unilateral biportal endoscopy/biportal endoscopic spinal surgery in the treatment of lumbar degenerative diseases: a bibliometric and visualization study. Orthop Surg. 2022;14:635–43. 35293686 10.1111/os.13216 PMC9002063

[12] Su BW Kim PD Cha TD. An anatomical study of the mid-lateral pars relative to the pedicle footprint in the lower lumbar spine. Spine. 2009;34:1355–62. 19478655 10.1097/BRS.0b013e3181a4f3a9

[13] Singhatanadgige W Jaruprat P Kerr SJ Yingsakmongkol W Kotheeranurak V Limthongkul W. Incidence and risk factors associated with superior-segmented facet joint violation during minimal invasive lumbar interbody fusion. Spine J. 2022;22:1504–12. 35447323 10.1016/j.spinee.2022.04.002

[14] Noren R Trafimow J Andersson GB Huckman MS. The role of facet joint tropism and facet angle in disc degeneration. Spine. 1991;16:530–2. 2052995 10.1097/00007632-199105000-00008

[15] Abumi K Panjabi MM Kramer KM Duranceau J Oxland T Crisco JJ. Biomechanical evaluation of lumbar spinal stability after graded facetectomies. Spine. 1990;15:1142–7. 2267608 10.1097/00007632-199011010-00011

[16] Lee WM You KH Kang MS Kim JH Park HJ. Oblique lumbar interbody fusion with selective biportal endoscopic posterior decompression for multilevel lumbar degenerative diseases. Asian Spine J. 2023;17:392–400. 36717091 10.31616/asj.2022.0227 PMC10151639

[17] Pao JL. Preliminary clinical and radiological outcomes of the “no-punch” decompression techniques for unilateral biportal endoscopic spine surgery. Neurospine. 2024;21:732–41. 38955542 10.14245/ns.2448376.188 PMC11224751

[18] Vanharanta H Floyd T Ohnmeiss DD Hochschuler SH Guyer RD. The relationship of facet tropism to degenerative disc disease. Spine. 1993;18:1000–5. 8367766 10.1097/00007632-199306150-00008

[19] Li W Han J Xin Q. Finite element mechanical analysis of ipsilateral approach and contralateral approach in unilateral bilateral endoscopic spine surgery. J Orthop Surg Res. 2023;18:979. 38124107 10.1186/s13018-023-04476-z PMC10734093

[20] Hu S Zhang J Zeng W. Imaging anatomy study related to unilateral biportal endoscopic lumbar spine surgery. Eur Spine J. 2024;33:4368–77. 38683383 10.1007/s00586-024-08270-1

[21] Park DK Weng C Zakko P Choi DJ. Unilateral biportal endoscopy for lumbar spinal stenosis and lumbar disc herniation. JBJS Essential Surg Techniques. 2023;13:e22.00020. 10.2106/JBJS.ST.22.00020 PMC10807897 38274147

[22] Son S Lee SG Kim WK Ahn Y. Advantages of a microsurgical translaminar approach (keyhole laminotomy) for upper lumbar disc herniation. World Neurosurg. 2018;119:e16–22. 29902597 10.1016/j.wneu.2018.06.004

[23] Sanderson SP Houten J Errico T Forshaw D Bauman J Cooper PR. The unique characteristics of “upper” lumbar disc herniations. Neurosurgery. 2004;55:385–9; discussion 389. 15271245 10.1227/01.neu.0000129548.14898.9b

[24] Tian D Zhu B Liu J. Contralateral inclinatory approach for decompression of the lateral recess and same-level foraminal lesions using unilateral biportal endoscopy: a technical report. Front Surg. 2022;9:959390. 36386540 10.3389/fsurg.2022.959390 PMC9661193

[25] Hwang JS Lee SH Jeong D. Far-lateral transforaminal unilateral biportal endoscopic lumbar discectomy for upper lumbar disc herniations. Neurospine. 2025;22:14–27. 40211509 10.14245/ns.2550058.029 PMC12010852

